# Proceedings of Societies

**Published:** 1867-04

**Authors:** 


### PROCEEDINGS OF SOCIETIES.

IT is not long since there was room in the journals for lengthly, even *verbatim* reports of the discussions of the societies then in existence. Now the case is different. So many societies have been formed, and so much is written, that we can not give all that attention that we would like. We have now on hand a phonographic report of the first meeting of the Ohio State Dental Society, a full report (our own) of the Boston meeting of the American Dental Association, also of the Conn. Valley Society, the Miss. Valley Association, etc., etc. We hope our brethren will be patient. We try to do the best we can. We can not always publish in the order received. Some are too long, some not ready for the printer, some are on subjects *now* agitating the profession, and are, therefore, hurried up. The REGISTER was started by the Miss. Valley Society, and it parted with it understanding that it would not cease to be its organ, and it came into our hands as, to some extent, the recognized organ of the Ohio College Association. With these facts in view, we impartially act as we think the cause of professional progress requires.

